# Do not stress, just differentiate: role of stress proteins in hematopoiesis

**DOI:** 10.1038/cddis.2014.560

**Published:** 2015-01-29

**Authors:** C Boudesco, T Rattier, C Garrido, G Jego

**Affiliations:** 1 INSERM UMR 866, « Equipe labellisée Ligue contre le Cancer » and Laboratoire d'Excellence LipSTIC, 7 Boulevard Jeanne d'Arc, 21000 Dijon, France; 2 University of Burgundy, Faculty of Medicine and Pharmacy, 7 Boulevard Jeanne d'Arc, 21000 Dijon, France; 3 CGFL, Centre de lutte contre le cancer GF Leclerc, Dijon, France

Hematopoiesis permits the constant regeneration of the blood system and is a permanent example of cell differentiation. Defects in its tight regulation can lead to either cell death or abnormal proliferation and may translate into multiple types of blood disorders, including leukemia. Heat shock proteins (HSPs), the expression of which is controlled by heat shock factors (HSFs, currently four known members),^1^ are a set of highly conserved proteins induced in response to a wide variety of physiological and environmental stress. HSP/HSF overexpression or mislocalization has been described in many cancers, particularly in hematology, and other diseases. Therefore, the involvement of HSFs/HSPs in the differentiation of cells from a hematopoietic origin is critical and is at the center of many investigations.^2, 3^ Here, we present newly identified mechanisms used by HSFs and HSPs to control the differentiation of several types of hematopoietic-derived cells.

For years, the common dogma has been that HSFs exclusively target heat shock elements (HSEs) present in the promoter of HSP genes to rapidly mount a protective response to heat-induced damage. However, recent genome-wide studies of the signaling pathways induced by the most studied HSF, HSF1, have demonstrated that this factor controls different transcriptional programs depending on the cellular stress experienced.^4^ Indeed, when HSF1 promoter binding was analyzed in the absence of heat shock, a very different profile was uncovered and included various sets of genes related to processes such as apoptosis, RNA splicing, and ubiquitination.^5^ Within the more extreme context of highly malignant tumors, HSF1 drives a unique transcriptional program that is distinct from heat shock and includes genes supporting oncogenic progression, cell-cycle regulation, and metabolism.^6^ Mendillo *et al.* demonstrated that HSF1 rewires the transcriptome during tumorigenesis, and we and others have similarly hypothesized that HSF1-dependent rewiring occurs during the course of another endogenous stress-like situation, namely, cellular differentiation.

To provide insight into a general view of HSF1 involvement in cell differentiation, we used an *in vivo* Hsf1^−/−^ mouse model and observed a significantly reduced percentage of myeloid cells in the bone marrow of *Hsf1*^−/−^ mice compared with wild-type mice.^7^ In addition, monocytes exhibited a reduced ability to differentiate *in vitro* into macrophages. We observed that the *SPI1/PU.1* gene that codes for the master myeloid transcription factor, contains several HSE-like sequences, including one particularly well-conserved sequence located in the second intron to which HSF1 binds during differentiation. Furthermore, HSF1 inhibition prevented an increase in *SPI1/PU.1* mRNA accumulation and thereby the differentiation of monocytes into macrophages.

Although an HSF1 binding site in the first intron of HSP27 and HSP90 beta was similarly described during hemin-induced erythroid differentiation of the myeloid K562 cell line, the functional repercussion on the differentiation process was not demonstrated.^8^ Furthermore, the putative binding of other HSF family members to nonpromoter regions has been previously described; >50% of HSF4 binding sites map to introns or exons in the genome, whereas only 5% map to promoter proximal regions.^9^ As described for HSF1 when binding to the IL-6 gene promoter,^10^ it is likely that HSF1 can facilitate the opening of the chromatin structure of the SPI1/PU.1 promoter through this intron-located HSE.

A recent study by Price and colleagues confirmed the role of HSF1 in other hematopoietic-derived cells, osteoclasts,^11^ which are multinucleated cells specialized in bone catabolism that originate from the myeloid lineage. Under appropriate *in vitro* culture conditions, osteoclasts can be derived from monocytes. Chai *et al.* were intrigued by the bone loss and increased osteoclast formation observed in mouse models treated with the geldanamycin-derived HSP90 inhibitor and anticancer drug 17-AAG^12^ and demonstrated an HSF-1-dependent increase in differentiation upon 17-AAG treatment.^11^ In contrast to monocyte-derived macrophage differentiation, no increase in HSF1 expression was observed upon 17-AAG treatment, suggesting the activation of HSF1 under stress. Although the exact mechanism governing this stress-induced osteoclast differentiation has not yet been identified, a very similar model to the one uncovered by Jego *et al.* has been proposed. Indeed, microphthalmia-associated transcription factor (MITF), a critical transcription factor for osteoclast formation and function, was strongly induced in an HSF1-dependent manner.^11^ MITF induction is hypothesized to be driven by the direct transcriptional action of HSF1 (either at the promoter level or at an intron-located regulatory site) or by the posttranslational stabilization of MITF via HSP70, which is strongly induced by 17-AAG.

It is somehow evident that HSF1 participates in the cell differentiation process not only by regulating the mRNA level of nonrelated HSP genes, but also by its well-known role in inducing the expression of HSP family members. The concept that cell differentiation needs a specific pattern of HSPs was first proposed in the early 1990s by Twomey *et al.*,^13^ who demonstrated that the pattern of HSP induction during myeloid differentiation of U937 cells differed from that observed when cells were subjected to heat shock, suggesting a unique program of HSP exploitation. Similarly, during hemin-induced erythropoiesis, some HSPs are more strongly expressed than after a heat shock, suggesting a specific role for HSPs in red blood cell formation.^8^ Under stress conditions, HSPs promote either the stability of selected proteins or their proteasomal degradation, thus contributing to cell survival. A similar role has been identified for HSPs within the context of cell differentiations.

For example, the HSP90 beta isoform binds to and protects cellular inhibitor of apoptosis protein-1 (cIAP-1) from proteasomal degradation, thereby allowing macrophage formation.^14^ At the late stage of erythropoiesis, the master transcription factor GATA-1 must be degraded. HSP27, an ATP-independent chaperone that binds ubiquitin and orchestrates the degradation of proteins such as IkBa, is phosphorylated and binds to GATA-1 to induce its ubiquitination and proteasomal degradation.^15^ Conversely, HSP70, the well-described role of which is to assist the folding of newly synthesized/misfolded polypeptides and to transport proteins across cellular membranes, protects critical transcription factors, such as SPI1/PU.1, from proteasomal degradation during macrophage differentiation. Given the early and transient nature of HSF1 activation during monocyte differentiation, HSF1 may first act directly like a sensor to initiate the differentiation process by inducing the transcription of SPI1/PU.1; HSF1 can then indirectly maintain the macrophage differentiation transcriptional program though the expression of HSP70, which stabilizes SPI1/PU.1.^7^

Partial caspase activation is required in several hematopoietic differentiation processes.^16, 17^ Protection of critical differentiation factors from caspase cleavage during this process is therefore necessary, and HSPs have a central role within this context. Ribeil *et al.*^18^ revealed that during erythropoiesis, HSP70 translocates into the nucleus and directly associates with the erythropoiesis master transcription factor GATA-1, thereby protecting it from caspase-3 cleavage. Given the previously described role of HSP27 in the proteasomal processing of GATA-1, HSP70, and HSP27 thus cooperate in the fine-tuning of terminal erythropoiesis through the regulation of GATA-1 content and activity.^15^ Confirming the essential role of HSPs in erythropoiesis, defaults in HSP post-translational modifications and/or in their subcellular localization lead to red blood cell pathologies, as evidenced in myelodysplastic syndromes (MDSs)^19^ and in beta-thalassemia major (*β*-TM).^20^

Erythroid cell dysplasia observed in MDS is due to a lack of terminal erythropoiesis and increased apoptosis. Frissan *et al.*^19^ demonstrated that a lack of cytosolic-nuclear HSP70 shuttling in MDS erythroblasts leads to GATA-1 cleavage and the subsequent inhibition of differentiation. Targeting this trafficking could serve as a new therapeutic approach in MDS anemia. Similarly, a defect in HSP70 nuclear localization was recently shown to be involved in the ineffective erythropoiesis observed in *β*-TM.^20^ The authors indicated that HSP70 accumulates in the cytoplasm of *β*-TM erythroblasts to chaperone excess free *α*-globin chains of hemoglobin, which are characteristic of the disease, and to limit the formation of its toxic aggregates. Thus, GATA-1 is no longer protected and erythroblasts die via apoptosis.

In conclusion, a body of strong evidence supports a role for HSFs and HSPs in hematopoietic cell differentiation (Figure 1). HSF and HSP actions occur at various levels, including (i) the transcription of HSPs and non-HSPs genes, (ii) the protection against proteolysis, and (iii) the regulation of cytosolic/nuclear shuttling of proteins.

However, as we gain insight into the regulation of cell differentiation, new questions arise. First, what is the interplay between the four HSF family members? Second, what is the role of HSPs other than the well-known HSP27, HSP70, and HSP90 proteins? Third, is the expression of other specific transcription factors finely tuned by HSFs either at promoter sites or at newly described intron-located *cis*-acting elements? The identification of these factors deserves considerable attention, as they are key inducers of cell differentiation.

## Figures and Tables

**Figure 1:**
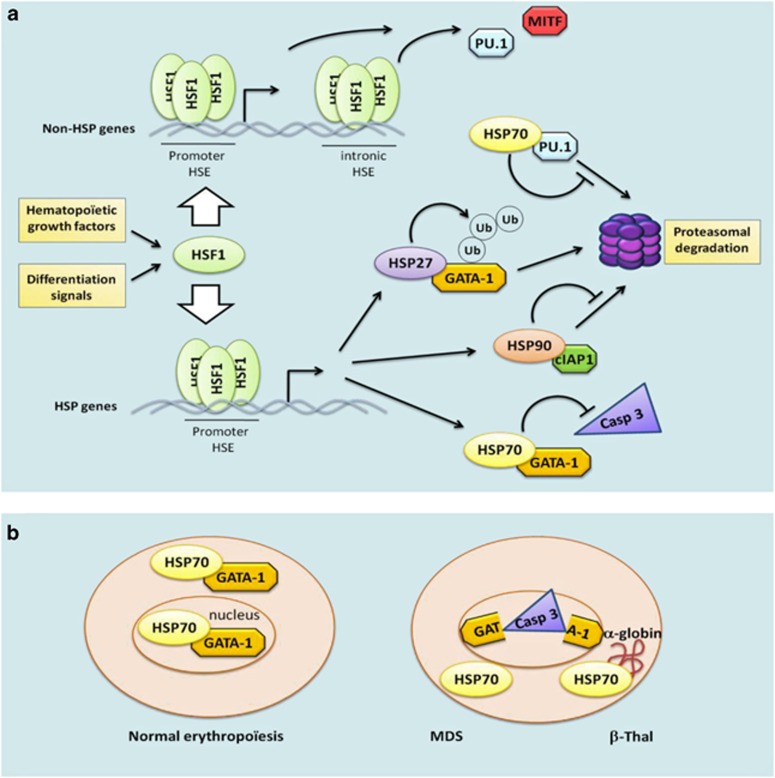
Mechanisms of action of HSF and HSPs in the control of expression and stability of hematopoïetic transcription factors. (**a**) Upon differentiation signals, HSF1 gets activated, trimerized, and binds HSE elements found in either the promoter or intronic regions of HSPs and non-HSPs genes. Critical transcription factors are then protected from caspase-3 cleavage (Casp 3), from proteasomal degradation, or conversely oriented towards proteolysis depending on the stage of differentiation and the needs of the cell. (**b**) In contrast to normal erythropoïesis, HSP70 cannot be translocated into the nucleus of erythroïd progenitors from MDS and from beta-thalassemia major (*β*-Thal) patients. GATA-1 is no more protected and subsequently cleaved by caspase-3

## References

[bib1] Akerfelt M et alNat Rev Mol Cell Biol 2010; 11: 545–555. 20628411 10.1038/nrm2938 PMC3402356

[bib2] Lanneau D et alPrion 2007; 1: 53–60. 19164900 10.4161/pri.1.1.4059 PMC2633709

[bib3] Mjahed H et alExp Cell Res 2012; 318: 1946–1958. 22652452 10.1016/j.yexcr.2012.05.012

[bib4] Vihervaara A, Sistonen L. J Cell Sci 2014; 127: 261–266. 24421309 10.1242/jcs.132605

[bib5] Page TJ et alMol Biosyst 2006; 2: 627–639. 17216044 10.1039/b606129j

[bib6] Mendillo ML et alCell 2012; 150: 549–562. 22863008 10.1016/j.cell.2012.06.031 PMC3438889

[bib7] Jego G et alLeukemia 2014; 28: 1676–1686.24504023

[bib8] Trinklein ND et alCell Stress Chaperones 2004; 9: 21–28. 15270074 10.1379/481.1 PMC1065302

[bib9] Fujimoto M et alJ Biol Chem 2008; 283: 29961–29970. 18755693 10.1074/jbc.M804629200 PMC2662063

[bib10] Inouye S et alJ Biol Chem 2007; 282: 33210–33217. 17766920 10.1074/jbc.M704471200

[bib11] Chai RC et alJ Biol Chem 2014; 289: 13602–13614. 24692538 10.1074/jbc.M113.530626 PMC4036365

[bib12] Price JT et alCancer Res 2005; 65: 4929–4938. 15930315 10.1158/0008-5472.CAN-04-4458

[bib13] Twomey BM et alClin Exp Immunol 1993; 93: 178–183. 8348742 10.1111/j.1365-2249.1993.tb07962.x PMC1554834

[bib14] Didelot C et alCell Death Differ 2008; 15: 859–866. 18239673 10.1038/cdd.2008.5

[bib15] de Thonel A et alBlood 2010; 116: 85–96.20410505

[bib16] Zermati Y et alJ Exp Med 2001; 193: 247–254. 11208865 10.1084/jem.193.2.247 PMC2193347

[bib17] Droin N et alFront Biosci (Landmark Ed) 2009; 14: 2358–2371. 19273205 10.2741/3383

[bib18] Ribeil JA et alNature 2007; 445: 102–105.17167422

[bib19] Frisan E et alBlood 2012; 119: 1532–1542.22160620

[bib20] Arlet JB et alNature 2014; 514: 242–246.25156257

